# Early Colonization with a Group of Lactobacilli Decreases the Risk for Allergy at Five Years of Age Despite Allergic Heredity

**DOI:** 10.1371/journal.pone.0023031

**Published:** 2011-08-01

**Authors:** Maria A. Johansson, Ylva M. Sjögren, Jan-Olov Persson, Caroline Nilsson, Eva Sverremark-Ekström

**Affiliations:** 1 Arrhenius Laboratories for Natural Sciences, Department of Immunology, The Wenner-Gren Institute, Stockholm University, Stockholm, Sweden; 2 Division of Mathematical Statistics, Department of Mathematics, Stockholm University, Stockholm, Sweden; 3 Department of Clinical Science and Education, Södersjukhuset, Karolinska Institutet and Sachs' Childrens Hospital, Stockholm, Sweden; Centre de Recherche Public de la Santé (CRP-Santé), Luxembourg

## Abstract

**Background:**

Microbial deprivation early in life can potentially influence immune mediated disease development such as allergy. The aims of this study were to investigate the influence of parental allergy on the infant gut colonization and associations between infant gut microbiota and allergic disease at five years of age.

**Methods and Findings:**

Fecal samples were collected from 58 infants, with allergic or non-allergic parents respectively, at one and two weeks as well as at one, two and twelve months of life. DNA was extracted from the fecal samples and Real time PCR, using species-specific primers, was used for detection of *Bifidobacterium (B.) adolescentis*, *B. breve*, *B. bifidum*, *Clostridium (C.) difficile*, a group of Lactobacilli (*Lactobacillus (L.) casei*, *L. paracasei* and *L. rhamnosus)* as well as *Staphylococcus (S.) aureus*. Infants with non-allergic parents were more frequently colonized by Lactobacilli compared to infants with allergic parents (p* = *0.014). However, non-allergic five-year olds acquired Lactobacilli more frequently during their first weeks of life, than their allergic counterparts, irrespectively of parental allergy (p* = *0.009, p* = *0.028). Further the non-allergic children were colonized with Lactobacilli on more occasions during the first two months of life (p* = *0.038). Also, significantly more non-allergic children were colonized with *B. bifidum* at one week of age than the children allergic at five years (p* = *0.048).

**Conclusion:**

In this study we show that heredity for allergy has an impact on the gut microbiota in infants but also that early Lactobacilli (*L*. *casei*, *L. paracasei*, *L. rhamnosus)* colonization seems to decrease the risk for allergy at five years of age despite allergic heredity.

## Introduction

During and after birth, the neonate is exposed to an array of microbes, which immediately start to colonize the skin and mucosal surfaces of the infant. The mode of delivery seems to play an important role in early-life colonization. During vaginal delivery, the neonate will be exposed to both vaginal and fecal flora, while a child delivered by caesarean section predominantly encounter skin bacteria [1]. The neonatal colonization pattern is further influenced by several post-natal environmental factors such as, the level of affluence, the number of siblings, the use of antibiotics and infant feeding [2]–[4]. As several bacterial species and bacterial components possess potent immunostimulatory capacities, these differential exposures may have implications for immune maturation [5]–[9]. Animal studies show that the gut microbiota composition is associated with immune development, maturation and function [8], [10] and also in humans associations between the early-life gut microbiota and mucosal and systemic immunity during the first year of life have been reported [11]–[13].

The rapid increase in allergic disease observed in the past decades is hypothesized to depend on microbial deprivation early in life [14], [15] Indeed, differences in the early infant gut microbiota in relation to development of allergic disease have been reported in some prospective studies [4], [16]–[18], but not in others [19]. For example, a reduced diversity [20] and lower counts of lactobacilli and bifidobacteria seem to characterize infants later developing allergy [4], [16]. On the contrary, early *Staphylococcus (S.) aureus* and *Clostridium (C.) difficile* colonization have been associated with development of allergy in some studies [16]–[18].

In most of these previous studies, a pronounced majority of the children have allergic heredity. Whether this influences the gut microbiota colonization, is not known, but recent studies indicate that host genetics is important for gut colonization [21]. Thus we studied whether parental allergy influenced infant gut colonization during the first year of life, as well as if the early-life gut microbiota was associated with future allergy development. Bacterial species investigated here: *B. adolescentis*, *B. bifidum*, *B. breve*, *C. difficile*, a group of Lactobacilli (*L. casei*, *L. paracasei* and *L. rhamnosus*) and *S. aureus* were included based on previously reported associations with allergic phenotypes during childhood. All children included in the study were born vaginally, exclusively breast fed during their first three months of life and none received antibiotics. Frequencies and relative quantities of above-mentioned bacteria were determined using real time PCR. The children were clinically followed to five years and diagnosed as allergic at the age of five only if the presence of allergen specific IgE and/or a positive skin prick test confirmed the allergic symptoms.

## Methods

### Study population

A total of 58 five-year old children and their mothers were selected for this study from a prospective birth-cohort (n = 281) previously described in detail elsewhere [22].

Parents were included only if self reported allergic status was confirmed with skin prick test (SPT) negative or positive results. The 58 infants, all born term (mean weeks 40, range 38–43), with normal birth weights (mean 3,7 kg, range 2,7–4,6), vaginally delivered and exclusively breast fed ≥3 months, were included based on availability of fecal samples at several occasions during infancy. Moreover, none of the infants received antibiotics the first three months of life. In total, 35 infants with allergic parents (60%) and 23 infants with non-allergic parents (40%) were included and demographic data of the individuals is displayed in Table 1. The subjects included in this study did not differ from the original cohort in respect to exposures, such as smoking, pets nor siblings (data not shown).

**Table 1:** Demographic data of the study population.

	All(n = 58)	Allergic Parents(n = 35)	Non-Allergic Parents(n = 23)	p-value
Maternal ageMedian (range)	31 (23–40)	32 (23–38)	30 (23–40)	0,359#
Maternal smokingn (%)	1 (1,7)	0 (0)	1 (1,7)	0,407£
Paternalsmoking n (%)	6 (10,3)	4 (6,9)	2 (3,4)	1,000£
Birth periodApril-Septembern (%)October-Marchn (%)	39 (67,2)19 (32,8)	24 (68,6)11 (31,4)	15 (65,2)8 (34,8)	1,000£
Siblingsmedian (range)	0 (0–5)	0 (0–5)	1 (0–3)	0,327#
Day care¶Attendance yes n (%)Start monthMedian (range)	45 (78,9)17 (12–25)	27 (79,4)17 (12–25)	18 (78,3)17,5 (12–20)	1,000 £0,638#
Furred pets at home n (%)	10 (5,8)	2 (5,7)	8 (34,8)	0,01£
IgE-mediated allergic disease§ at five n (%)	20 (34,5)	16 (45,7)	4 (17,4)	0,047£

# Mann Whitney Rank Sum test.

£ Fishers Exact test.

¶ Missing data for one individual.

§ IgE-mediated disease according to the WAO nomenclature [23].

The study was approved by the Human Ethics Committee at Huddinge University Hospital, Stockholm (Dnr 75/97, 331/02), and the parents provided informed verbal consent. Families expecting a child were asked by the midwife at the maternity ward if they were interested in participating in the study. If so, the pediatrician (C.N.) in charge contacted them, gave further information and invited them to a seminar on allergies. If still interested, appointments were made for blood sampling of the parents, when approval of their participation was documented. No written documentation of the participants informed approval was required, which was agreed to by the Human Ethics Committee.

### Skin prick testing and determination of allergen specific IgE in circulation

At age five, SPT were performed against food and inhalant allergens according to the manufacturer's instruction (ALK, Copenhagen, Denmark). The SPT included food allergens: egg white (Soluprick weight to volume ratio 1/1000), cod (Soluprick 1/20), peanut (Soluprick 1/20) cow's milk (3% fat, standard milk), and soybean protein (Soja Semp; Semper AB, Stockholm, Sweden). SPTs were also performed for inhalant allergens: cat, dog, *Dermatophagoides farinae*, birch and timothy (Soluprick 10 Histamine Equivalent Prick test). Histamine chloride (10 mg/mL) and the allergen diluent were the positive and negative controls, respectively. The SPT was considered positive if the wheal diameter was ≥3 mm after 15 minutes. All parents were skin prick tested against the same inhalant allergens as the children but also against horse, rabbit, and mugwort.

Serological analysis of specific IgE, to the same allergens tested with SPT, was performed using ImmunoCAP (Phadia AB, Uppsala, Sweden). Samples with allergen-specific levels of ≥0.35 kU_A_/L were considered positive.

### Clinical evaluation and classification of IgE-mediated allergic disease

Subjects were followed from birth to five years of age and were clinically examined by the same pediatrician (C.N.). At five years of age, the children were classified as allergic (n = 20) if at least 1 SPT was positive (≥3 mm) and/or if specific IgE to at least 1 of the selected allergens was ≥0.35 kU_A_/l together with allergic symptoms at five years of age [23]. Children with negative SPT together with negative allergen specific IgE and not having any allergic symptoms at five years of age were classified as non-allergic (n = 19). In addition to the allergic and non-allergic children, fifteen children were non-sensitized but had symptoms such as eczema (n = 11) or asthma (n = 4) thus they were excluded in the five year analyses of infant microbiota in relation to IgE-mediated allergy at five together with children lost in the follow up (n = 4).

### Detection of bacterial species in fecal samples

The methods used in the study are previously published in detail [4]. Briefly, infant fecal samples were collected at 1 and 2 weeks and at 1, 2 and 12 months of age. Maternal fecal samples were collected in connection to the delivery. The samples were brought to the hospital on ice and stored at −70°C until analysis. DNA from the fecal samples was extracted using the Qiamp DNA Stool Mini Kit™ protocol increasing the bacterial DNA of the human DNA (Qiagen, Hilden, Germany). Extracted nucleic acid concentration was measured with Bio-Rad Smartspec (Bio-Rad Laboratories, Hercules, CA, USA) at 260 nm using Bio Rad trUView Disposable Cuvettes (Bio-Rad Laboratories).

Real time PCR was used for analyses of bacterial DNA, using SYBR Green chemistry with primer pair sequences and concentrations previously published. Primer pairs used targeted *C. difficile*, *B. adolescentis*, *B. bifidum*, *B. breve*, a group of Lactobacilli (*L. casei*, *L. paracasei*, *L. rhamnosus*) [4] and *S. aureus* [24]. *L. casei*, *L. paracasei*, *L. rhamnosus* were detected with one primer pair and will be referred to as “Lactobacilli” from now on.

Reference bacterial DNA, purchased from LGC Standards (Borås, Sweden) and Biotechon Diagnostics (Potsdam, Germany) was used as standards and positive controls.

SYBR Green real-time PCR was performed using 96-well detection plates in ABI prism 7000 (Applied Biosystems, Stockholm, Sweden). The Absolute Quantification protocol in 7000 SYSTEM software version 1.2.3f2 (Applied Biosystems) was used together with a standard curve ranging 5 ng to 50 fg. All samples were analyzed in triplicates with each well containing 2xPower SYBR Green Mastermix (Applied Biosystems), primer pairs (MWG-Bioteck, Edersburg, Germany), sample (DNA) and water. Triplicates with C_T_ values above 35 were considered negative avoiding detection of false positives. Calculation of amount specific bacterial DNA was performed from the standard curve using The Absolute Quantification protocol in 2000 SYSTEM software. Relating the amount of specific bacterial DNA to the total amount of nucleic acids in each sample gives percentage of specific bacterial DNA/total nucleic acids and is referred to as relative amounts in percentage and the limit of detection was 5×10^−6^%.

### Statistics

Statistical analyses were performed at the Division of Mathematical Statistics, Stockholm University. Fisher's exact test was used to evaluate differences in frequencies between the groups of infants with allergic parents versus non-allergic parents, and also between children who developed allergy as compared to the ones remaining non-allergic. To assess differences in the relative amounts of the detectable bacteria the Mann-Whitney Rank Sum test was applied. Additionally, to investigate differences in the number of occasions with the bacteria the first two months, Mann Whitney Rank Sum test was used.

To assess the microbiota with respect to both allergic heredity and allergic disease at age five, Fisher's exact test was used for the frequencies together with Kruskal Wallis Rank Sum test comparing the relative amounts between all groups. Where significant differences between all groups were obtained, further pair wise statistical analyses were performed as above using Fisher's exact test and Mann-Whitney Rank Sum test. Many statistical tests were performed, and no correction for multiple testing was done, potentially generating false significances.

## Results

### Infants with non-allergic parents are frequently colonized by Lactobacilli

**Figure 1:**
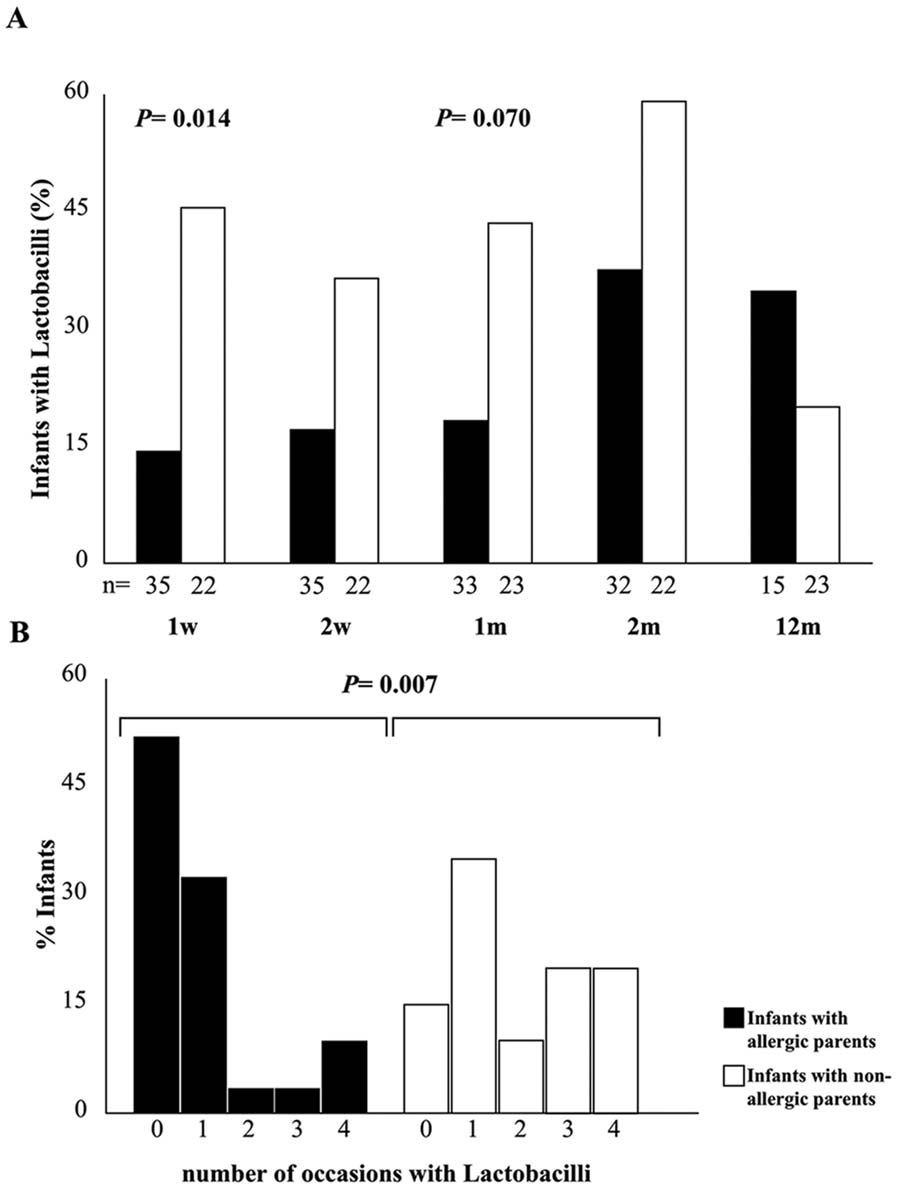
Intestinal colonization with Lactobacilli in infants with allergic and non-allergic parents respectively. The y-axis shows percentage infants colonized at the different time points (**A**) and percentage infants colonized 0, 1, 2, 3 or 4 occasions the first two months of life in (**B**).

Infants born to non-allergic parents (n = 23) were significantly more often colonized by the Lactobacilli at one week than infants born to allergic parents (n = 35) (Fig. 1A). This pattern was similar at the other investigated occasions during the first two months of life, although not statistically significant (Fig. 1A). The number of occasions, having Lactobacilli during the first two months, was significantly higher among infants with non-allergic parents (Fig. 1B). Furthermore, the relative amounts of the Lactobacilli were significantly higher or tended to be higher in infants with non-allergic parents at one and two weeks as well as at one and two months of age (p* = *0.013, p* = *0.065, p* = *0.036 and p* = *0.058 respectively) (data not shown).

In addition, the relative amounts of *B. bifidum* were significantly higher in infants with non-allergic parents at one week of age (p* = *0.044) (data not shown), but neither the frequencies (Table 2) nor the amounts (data not shown) of the other species investigated, differed with parental allergic status.

**Table 2:** Frequencies of bacterial species in infants with allergic and non-allergic parents, respectively.

	All %(n = 48)	Allergic mothers % (n = 30)	Non-Allergic mothers % (n = 18)
**Lactobacilli**	71	67	78
***B. adolescentis***	79	73	89
***B. breve***	10	17	0
***B. bifidum***	44	37	56
***C. difficile***	0	0	0
***S. aureus***	13	13	11

Allergic parents (A parents), Non-allergic parents (NA parents).

*C. difficile* was only detected in two infants during the first two months of life whereas half of all children were colonized at twelve months of age (Table 2). The opposite pattern was observed regarding *S. aureus*, with many infants colonized early but few at twelve months of age (Table 2).

At twelve months of age the infants with allergic parents seemed more frequently colonized by all the species investigated, compared to the children with non-allergic parents, although not statistically significant (Fig. 1A, Table 2).

### The early-life microbiota associates with IgE-mediated allergic disease at five years of age

At the age of five, 20 children were considered allergic (SPT ≥3 mm and/or allergen-specific IgE ≥0,35 kU/l together with allergic symptoms) while 19 children fulfilled the criteria of being non-allergic, as they showed no allergic symptoms and were non-sensitized.

**Figure 2:**
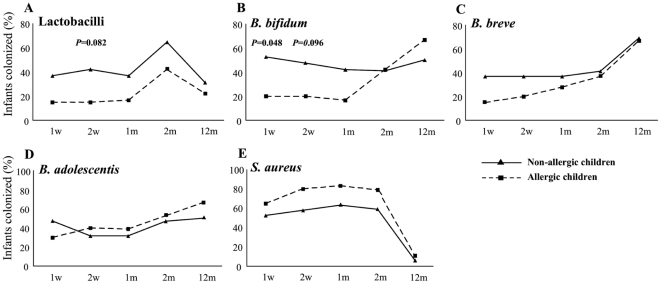
Early life gut microbiota composition associates with allergic disease at age five. The y-axis shows the percentage, colonized with (**A**) Lactobacilli, (**B**) *B. bifidum*, (**C**) *B. breve*, (**D**) *B. adolescentis* and (**E**) *S. aureus*. Solid line; non-allergic children n = 16–19. Dashed line; allergic children n = 18–20 (1 v–2 m), n = 9 (12 m).

Frequencies of the different investigated species in relation to allergy are depicted in Figure 2. The Lactobacilli tended to be more commonly detected in early fecal samples from children who were non-allergic at five years of age than in children who had developed allergy (Fig. 2A). Moreover, the non-allergic children were colonized with Lactobacilli on more occasions during the first two months of life (p* = *0.038, data not shown) than the allergic group. However, the relative amounts did not differ between the groups (data not shown).

Additionally, the pattern of colonization was similar for *B. bifidum* and *B. breve* (Fig. 2B–C). Non-allergic children were significantly more colonized with *B. bifidum* at one week of age, with a similar tendency at two weeks of age (Fig. 2B). The colonization with *B. breve* or *B. adolescentis* did not differ with respect to frequencies (Fig. 2C–D) or relative amounts (data not shown) at any time point between the two groups. *S.aureus* frequencies, early in life, seemed higher in the allergic group but not statistically significant (Fig. 2E). *C. difficile* was only detected in two infants and did not differ between the allergic and non-allergic group neither the first two months nor at 12 months of age (data not shown). At twelve months of age the frequencies of all species investigated were similar in both groups (Fig. 2A–E).

### Early Lactobacilli colonization decreases the risk for allergy at five years of age despite allergic heredity

We further sub-grouped the children considering both parental allergy and IgE-mediated allergic disease at five. Among the children with allergic parents, 16 had developed IgE-mediated allergy and 8 remained non-allergic at five years of age. In the group of children with non-allergic parents, 11 remained non-allergic at five years, while 4 children were allergic. These 4 children were excluded from following analyses, as they were too few subjects for a reliable statistical outcome.

**Figure 3:**
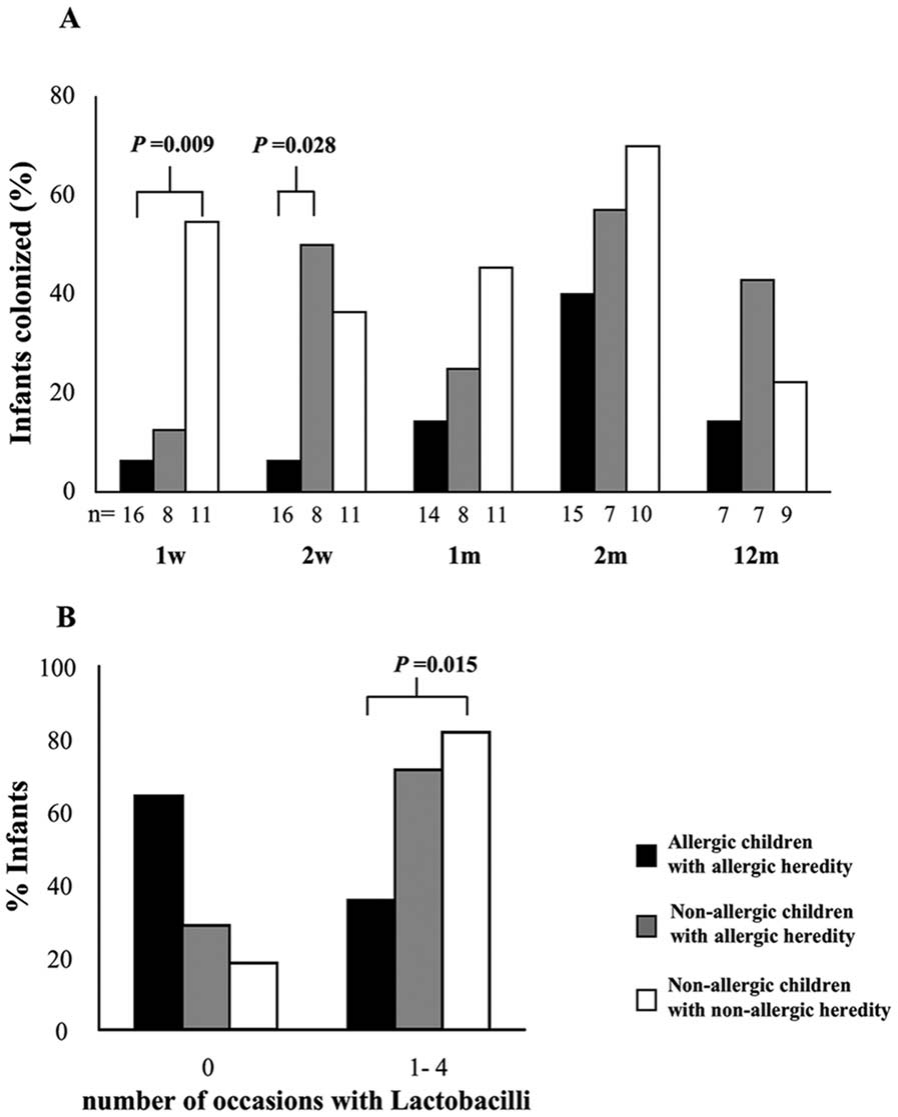
Colonization with Lactobacilli the first year of life, sub grouped according to both parental allergy and allergy at five years of age. Proportion of infants, in percentage, the different time points investigated in (A). Percentage infants colonized 0 or 1–4 occasions the first two months of life in (B).

When analyzing the infant gut microbiota of children with allergic parents, we observed that the frequency of colonization at two weeks differed between allergic and non-allergic children with allergic parents (Fig. 3A). In consequence, among children with allergic parents, the children colonized with Lactobacilli at two weeks of age were less likely to be allergic at age five.

Also, non-allergic children were colonized with Lactobacilli at more occasions during the first two months of life (Fig. 3B).

A similar colonization pattern in relation to parental allergy and allergic disease could also be seen for *B. bifidum*, although non-statistically significant (data not shown). More than half of the children were colonized with *S. aureus*, already at 1 week of age, regardless of parental allergy or allergy development.

As previously, at twelve months of age the species investigated were similar in all groups (data not shown).

### The maternal gut microbiota is not influenced by allergy

As the early infant microbiota could depend on exposures during delivery we investigated the maternal fecal microbiota at the time around delivery. The maternal frequencies are shown in Table 3. Among all the mothers investigated (n = 48), *B. adolescentis* was the most commonly species detected, followed by the Lactobacilli. *S. aureus* colonization was rare and *C. difficile* was not detected in any of the maternal samples analyzed. The presence and the relative amounts of the analyzed bacteria did not differ significantly between non-allergic and allergic mothers. When correlating paired maternal and infant one-week samples (n = 47) we found significant correlations for *B. breve* (p* = *0.030) and *B. bifidum* (p* = *0.002) (data not shown), but not for any of the other investigated bacteria.

**Table 3:** Frequencies of species investigated among allergic and non-allergic mothers.

Occasion	1 week			2 weeks			1 month			2 months			12 months		
	All n = 57	Aparents n = 35	NA parents n = 22	All n = 57	Aparents n = 35	NA parents n = 22	All n = 56	A parents n = 33	NA parents n = 23	All n = 54	Aparents n = 32	NA parents n = 22	All n = 38	Aparents n = 23	NA parents n = 15
*B. adolescentis* (%)	40	31	55	30	31	27	25	21	30	41	38	45	58	61	53
*B. breve* (%)	28	29	27	28	26	32	30	33	26	33	38	27	61	70	47
*B. bifidum* (%)	33	26	45	32	29	36	32	30	35	43	50	32	58	70	40
*C. difficile* (%)	0	0	0	4	3	5	4	3	4	4	3	5	53	61	40
*S. aureus* (%)	63	63	64	72	74	68	79	79	78	76	75	77	8	13	0

## Discussion

Early in life the immune system is immature and requires stimuli from the environment, such as microbial exposures, to mature properly. The gut microbiota composition has previously been reported to differ during infancy prior to the development of allergic disease, implying a role of the gut microbiota in promoting tolerance to harmless antigens through education of the immune system.

In this study we show that, colonization is related to both allergic heredity and IgE-mediated allergic disease at five years of age in a well-characterized cohort. We clearly demonstrate that the proportion of infants colonized with Lactobacilli early in life, is significantly higher in infants with non-allergic parents than infants with allergic parents (Fig. 1A). Further, infants with non-allergic parents are colonized with Lactobacilli more often (Fig. 1B) and have larger relative amounts of both Lactobacilli and *B. bifidum* in their fecal samples than infants with allergic parents. To our knowledge, this is the first time that colonization during infancy is shown to be associated with parental allergic status. In previous studies [4], [18], [19] most of the parents, especially the mothers, were allergic and the authors could not compare with children having non-allergic parents.

In this context, it is interesting to note that levels of endotoxin in house dust are reported to be lower in families with allergic parents [25]. This can potentially reflect the number of bacteria that the infant encounters as it has previously been shown that higher endotoxin levels are associated with an increased number of bifidobacteria species in infant feces [4].

In addition, we are able to confirm previously published results from our group [4] that among five-year old children, non-allergics are significantly more often colonized with Lactobacilli, compared to children developing IgE-mediated allergic disease (Fig. 2A). Recently, Penders et al also reported similar results in a large cohort, including several hundred children, evaluated at a single early occasion [26]. Furthermore the proportion of infants having *B. bifidum* is higher among the non-allergic children throughout the first months of life (Fig. 2B) and in line with Ouwehand et al [27]. They report that *B. bifidum* was less often detected in already allergic children compared to their age-matched non-allergic controls. *B. bifidum* has previously been correlated with IgA in saliva at 12 months of age [13] which potentially could protect sensitized infants from developing allergic symptoms as secretory IgA is suggested to block the allergen and IgE interaction [28].

In our study *S. aureus* was more commonly detected among children developing allergy, although not statistically significant (Fig. 2E) thus the relation between *S. aureus* and allergy development remains unclear. However our data regarding *S. aureus* as a frequent colonizer during infancy is in line with previously published results by Adlerberth et al [29].

*C. difficile* was only detected in two infants the first two months of life, whereas approximately half of the children harbored *C. difficile* at twelve months of age and no association with allergy development was observed. All infants were vaginally delivered, exclusively breast-fed and none received antibiotics during the first three months of life, which may explain the low prevalence of *C. difficile* as it is more prevalent in caesarean delivered and formula fed infants [19].

Generally the kinetics of colonization postnatally seems to differ in the non-allergic children compared to the allergic children where the children developing allergy seems to have a delayed colonization in early infancy (Fig. 2). On the contrary, at twelve months of age the groups of children were similar in the frequencies of the different species investigated, suggesting that it is the early microbiota exposure that is of importance for future development of allergic disease.

Importantly, we were further able to demonstrate that among children with allergic parents, infants colonized with Lactobacilli were less likely to be allergic at the age of five (Fig. 3A). This might suggest that colonization with Lactobacilli protects against development of allergic disease despite parental allergy. Supporting this interpretation are data from studies where probiotic supplementation early in life, with different species of Lactobacilli, reduced clinical outcomes such as eczema and/or IgE sensitization [30]–[31]. Recently, administration of *L*. *rhamnous* protected against allergen-induced allergic disease in a pig model [32]. Also, a reduction of specific IgE accompanied by increased specific IgG following Lactobacilli administration to patients compared to placebo-treatment has been reported [33]. However there are also studies reporting no beneficial effects on neither allergic symptoms nor IgE sensitization following probiotic supplementation [34]–[36].

When investigating the maternal fecal microbiota we found that the allergic and non-allergic mothers were similarly colonized with regards to the bacteria analyzed. A correlation between maternal and infant *B. breve* and *B. bifidum* colonization was observed the first week of life. Unfortunately, neither breast milk samples nor vaginal swabs were collected thus we cannot exclude that the infants are differently exposed. Grönlund et al has reported lower counts of bifidobacteria in breast milk from allergic mothers compared to non-allergic mothers [37].

The number of children investigated here equals most of the other similar studies, though we should acknowledge the small sample size when subgrouping, thus future studies with more subjects are needed to elucidate the actual role of the microbiota in relation to allergy development. However, results from a recent study by Penders et al [26] investigating a cohort with more than 600 children supports our findings. Also, many statistical tests were done, potentially generating false significances. Strengths of this study are that all infants were born term, vaginally delivered and breast fed the first three months of life, factors known to influence the microbiota composition. Further, we have investigated the gut microbiota at several occasions in the same children. Also, we have included children with both allergic and non-allergic parents, enabling us to evaluate the association between parental allergy and early colonization. Finally, we evaluated early gut flora in relation to allergy, based both on SPT and/or specific IgE and allergic symptoms at five years of age. Many other studies have eczema and wheezing/asthma as an outcome but it is known that approximately half of the children with those symptoms are not IgE sensitized. To consider IgE sensitization in combination with allergic symptoms gives a better classification of IgE mediated allergic disease.

In this study we clearly demonstrate that parental allergy has an impact on the infant gut microbiota, and that early Lactobacilli (*L*.*casei*, *L. paracasei, L. rhamnosus)* colonization seems to decrease the risk for allergy at five years of age despite allergic heredity. Even though previous results regarding allergy development and early probiotic supplementation are contradictory, our findings suggest that the naturally acquired gut microbiota has an impact on allergic disease. The exact mechanisms on how the different species interact with and influence the immune system are unclear and require further investigation.
